# Comparison of Bilevel Volume Guarantee and Pressure-Regulated Volume Control Modes in Preterm Infants

**DOI:** 10.3390/children10101603

**Published:** 2023-09-26

**Authors:** Şehribanu Işık, Fuat Emre Canpolat, Gülsüm Kadıoğlu Şimşek, Ömer Ertekin, Hayriye Gözde Kanmaz Kutman

**Affiliations:** Department of Neonatology, University of Health Sciences, Ankara City Hospital MH5, Çankaya, Ankara 06800, Turkey; fuatemre.canpolat@saglik.gov.tr (F.E.C.); g.kadioglusimsek@saglik.gov.tr (G.K.Ş.); omer.ertekin1@saglik.gov.tr (Ö.E.); hayriyegozde.kanmazkutman@sbu.edu.tr (H.G.K.K.)

**Keywords:** bilevel volume guarantee, pressure-regulated volume control, preterm, mechanical ventilation

## Abstract

The present study aimed to compare the bilevel volume guarantee (VG) and pressure-regulated volume control (PRVC) modes of the GE^®^ Carescape R860 model ventilator and test the safety and feasibility of these two modes in preterm neonates. Infants who were less than 30 weeks of gestational age were included. After randomization, initial ventilator settings were adjusted for each patient. After the first 2 h of ventilation, the patients were switched to the other ventilator mode for 2 h. The ventilator parameters, vital signs, and blood gas values were evaluated. The study included a total of 28 patients, 14 in the PRVC group and 14 in the bilevel VG group. The mean birth weight was 876 g (range: 530–1170) and the mean gestational age was 26.4 weeks (range: 24–29). The patients’ peak inspiratory pressure (PIP_2_ and PIP_3_) was lower after ventilation in bilevel VG mode than in PRVC mode (13 vs. 14 cmH_2_O, respectively; paired samples *t*-test, *p* = 0.008). After 2 h of bilevel VG ventilation, the mean heart rate decreased from 149/min to 140/min (*p* = 0.001) and the oxygen saturation increased from 91% to 94% (*p* = 0.01). Both the PRVC and bilevel VG modes of GE ventilators can be used safely in preterm infants, and bilevel VG mode was associated with more favorable early clinical findings. Studies including more patients and comparing with other modes will clarify and provide further evidence on this subject.

## 1. Introduction

One of the most important steps in reducing neonatal morbidity is to prioritize noninvasive respiratory support and avoid invasive mechanical ventilation. However, the latter is used as rescue therapy in infants with severe respiratory distress. According to US Neonatal Research Network data for 2018, 84.3% of infants at <29 weeks of gestation received conventional mechanical ventilation support during their stay in the NICU [1].

Rapid advances in ventilator technology have resulted in numerous basic and complex respiratory support modes. To understand how a mechanical ventilator works, one must be knowledgeable about the features of these specific modes, how they interact with an alert and spontaneously breathing infant, and mode definitions specific to the ventilator in the unit [2,3].

At present, tidal volume-targeted synchronized ventilation modes are most recommended for ventilating preterm infants. Tidal volume-targeted synchronized ventilation has been shown to provide shorter ventilatory support duration and lower rates of adverse effects such as pneumothorax, hypocarbia, severe intracranial hemorrhage, mortality, and bronchopulmonary dysplasia (BPD) [4,5,6,7].

Along with the increase in available ventilator types and modes, ventilator modes have become more complex, and the parameters that were previously entered and adjusted by the clinician are now automatically adjusted by the device. Bilevel volume guarantee (VG) and pressure-regulated volume control (PRVC) modes are examples of this adaptive target scheme [2]. In an adaptive target scheme, the ventilator uses breath-to-breath feedback to continuously adjust the delivered pressure to achieve the target tidal volume (V_T_) [8,9].

As experience and evidence regarding the use of new tidal volume-targeted devices and modes in neonatal intensive care units (NICUs) are limited, there is a need for safety and efficacy studies and comparative trials of these modes. The present study aimed to compare the bilevel VG and PRVC modes of the GE^®^ Carescape R860 model ventilator and test the safety and feasibility of these two modes in preterm neonates.

## 2. Materials and Methods

This study was conducted in the NICU of Ankara City Hospital between January 2020 and June 2021, serving as a referral for tertiary care. This study was designed as a prospective, randomized crossover study. Infants who were less than 30 weeks of gestational age, required intubation and mechanical ventilation in the first 24 h of life, received surfactant at least once, and were clinically stable at postnatal 24 h were included in the study. Patients whose families did not provide consent, who had major congenital anomalies at admission, or who had congenital heart disease were excluded. Patients who met the selection criteria were randomized after the first surfactant administration to crossover ventilation with bilevel VG and PRVC for 2 h each using the sealed envelope method with grouping by weight.

- Group 1 (PRVC): Bilevel VG mode first, PRVC mode second, then continued with PRVC
- Group 2 (bilevel VG): PRVC mode first, bilevel VG mode second, then continued with bilevel VG.

In block randomization, weight stratification categories were determined as 500–750 g, 751–1000 g, and 1001–1250 g. As blinding was not possible due to the nature of the study, the sealed envelope method was used to help ensure balance and reduce bias. 

After group assignment, the infants were monitored with the designated ventilation mode. The following ventilator parameters were set in PRVC mode: FiO_2_, positive end-expiratory pressure (PEEP), pressure support (PS), V_T_, respiratory rate, inspiratory time (Tins), inspiratory pressure minimum (Pmin) and maximum (Pmax). In bilevel VG mode, FiO_2_, V_T_, PEEP, respiratory rate, Tins, and Pmax were set. V_T_ was set to 5 mL/kg and the target blood gas values were pH 7.25–7.35, PaCO_2_ 45–55 mmHg, and PaO_2_ 50–70 mmHg.

### 2.1. Operating Principles of the Ventilation Modes

In SIMV PRVC mode, the ventilator delivers synchronized pressure-regulated, volume-controlled breaths at the set respiratory rate. For each mechanical breath, the ventilator adjusts the inspiration pressure to the lowest pressure needed to provide the target V_T_. All spontaneous efforts trigger pressure-assisted breaths. 

The ventilator provides volume-controlled ventilation for 10 s or two breaths (whichever is longer when the mode is initiated) to determine the patient’s lung compliance. Inspiratory pressure is determined according to lung compliance and used for subsequent breaths. 

When adjusting inspiratory pressure, the ventilator uses PEEP + Pmin as the lower limit and Pmax—5 cmH_2_O as the upper limit. The difference in inspiratory pressure between breaths does not exceed ±3 cmH_2_O. If a high airway pressure alarm is activated for the current breath, the target pressure for the next breath is reduced by 0.5 cmH_2_O.

In Bilevel VG mode, if the patient initiates a breath at the PEEP level, a pressure-supported breath is provided at the set PS. The ventilator switches between the PEEP and minimum pressure to maintain the target V_T_ according to the set respiratory rate and Tins values. As in PRVC mode, the ventilator determines lung compliance by providing volume-controlled ventilation over 10 s or two breaths (whichever is longer when the mode is initiated) and this is used to determine inspiratory pressure for subsequent breaths. Inspiratory pressure limits and adjustments are also the same as in PRVC mode. 

**Figure 1:**
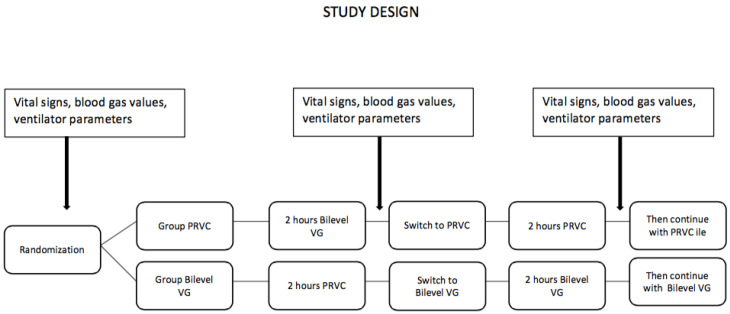
Study design.

All patients were monitored with a GE^®^, Chicago, IL, USA, Carescape R860 model ventilator. After randomization, initial ventilator settings were adjusted for each patient and recorded as the starting point. Respiratory rate, heart rate, and pulse oximetry (SpO_2_ 90–94%) values were continuously monitored in all patients. After the first 2 h of ventilation, the patients were switched to the other ventilator mode for 2 h. Ventilator parameters, vital signs, and blood gas values were evaluated at the beginning of the study, after the first ventilation mode, and after the second ventilation mode (Figure 1). Infants with oxygen requirements greater than 35% received a second dose of surfactant 8–12 h after the first dose. 

### 2.2. Definitions and Neonatal Outcomes

Gestational age was determined based on the mother’s last menstrual period, first-trimester prenatal ultrasound (US) data, or estimated postnatally using the New Ballard Score [10]. Respiratory distress syndrome (RDS) was defined as early postnatal symptoms of respiratory distress accompanied by tachypnea, retraction, grunting, and cyanosis and supported by blood gas and chest X-ray findings [11,12,13].

In accordance with unit protocols, surfactant therapy for RDS was administered prophylactically at NICU admission or as early rescue therapy in the first two hours according to the following criteria: as per the recommendations of the Turkish Neonatal Society, preterm infants at gestational age < 26 weeks that did not receive antenatal steroid therapy and preterm infants that required intubation in the delivery room received prophylactic surfactant; infants with signs of RDS findings and FiO_2_ requirement > 40% received early rescue surfactant therapy. All patients in the study received surfactant (poractant alfa 200 mg/kg) within the first 2 h of NICU admission and were monitored using GE^®^, Carescape R860 model ventilators. 

Noninvasive ventilation techniques and less invasive surfactant administration methods are encouraged in all patients with a good respiratory drive however patients were intubated and mechanically ventilated if they fulfilled the following criteria. Hypoxemia despite FiO_2_ requirement ≥ 60%, respiratory acidosis (pH < 7.2) under PEEP ≥ 7 cm H_2_O, and recurrent apnea.

BPD in a preterm infant < 32 weeks of gestational age is defined as radiographically confirmed persistent parenchymal lung disease and is classified according to oxygen requirement at postmenstrual 36 weeks as follows [14,15]:

- Mild BPD, nasal cannula oxygen < 2 L/min, FiO_2_ ≥ 21;
- Moderate BPD, nasal cannula oxygen > 2 L/min or nCPAP or NIPPV, FiO_2_ ≥ 21;
- Severe BPD, invasive PPV, FiO_2_ ≥ 21.

PDA refers to the nonclosure of the ductus arteriosus after the first 72 h of life. The diagnosis of hemodynamically significant PDA is made by demonstrating high-volume flow through the PDA on echocardiography. The first choice of medical treatment for PDA in our clinic is ibuprofen in two doses, 10 mg/kg/day followed by 5 mg/kg/day, administered 24 h apart [16].

ROP screening is performed in all infants born at a gestational age of <34 weeks and a birth weight of <1700 g, as well as infants with a gestational age of ≥34 or a birth weight of >1700 g who received cardiopulmonary supportive treatment or is considered by the attending clinician to be at risk of ROP. The first ophthalmological examination is performed at postnatal 4 weeks. In our unit, the criteria specified by the multicenter ETROP (Early Treatment for Retinopathy of Prematurity) study group are used in the application of laser photocoagulation for ROP [17,18].

Proven sepsis is defined as the presence of clinical and laboratory findings consistent with sepsis and a demonstrated causative pathogen. Clinical sepsis is defined as the presence of clinical and laboratory findings consistent with sepsis for which a causative pathogen could not be demonstrated [19,20]. Intraventricular hemorrhage (IVH) is evaluated using bedside cranial ultrasound (US) and staged according to the Papile classification [21].

Data regarding the patients’ survival to discharge, any stage of BPD, PDA requiring medical treatment, any stage of ROP, grade 3–4 IVH, and clinical or proven sepsis was recorded from the patients’ records. 

Follow-up ventilation management and other supportive treatments were provided by the attending neonatologist in accordance with unit protocols. In our unit, the feeding and care of preterm infants and the diagnosis and management of RDS, BPD, patent ductus arteriosus (PDA), and the retinopathy of prematurity (ROP) were carried out in accordance with Turkish Neonatology Society guidelines [13,14,16,17,19,22].

Written informed consent was obtained from the patients’ families. The study was approved by the Ankara City Hospital Ethics Committee (E1-19-198) in accordance with the Helsinki Declaration and prospectively registered at clinicaltrials.gov (accessed on 12 May 2019) (NCT04191239).

### 2.3. Statistical Analysis

The data were analyzed using SPSS for Windows. Descriptive statistics are shown as frequency and percentage, mean and standard deviation, or median and range. A paired samples *t*-test was used for comparisons of repeated measures within subjects and the Mann–Whitney U test was used for comparisons between the two different groups. Statistical significance was accepted at *p* < 0.05. 

## 3. Results

The study included a total of 28 patients, 14 in the PRVC group and 14 in the bilevel VG group. The mean birth weight was 876 g (range: 530–1170) and the mean gestational age was 26.4 weeks (range: 24–29). The characteristics of the patients are shown in Table 1. 

The patients’ peak inspiratory pressure (PIP_2_ and PIP_3_) was lower after ventilation in bilevel VG mode than in PRVC mode (13 vs. 14 cmH_2_O, respectively; paired samples *t*-test, *p* = 0.008). After 2 h of bilevel VG ventilation, the mean heart rate decreased from 149/min to 140/min (*p* = 0.001) and the oxygen saturation increased from 91% to 94% (*p* = 0.01). There were no significant differences in mean airway pressure, respiratory rate, or blood gas values. 

As shown in Table 2, there was no difference between the groups in terms of clinical outcomes. Both groups had similar rates of PDA, BPD, ROP, sepsis, and mortality. Two patients in the bilevel VG group and three patients in the PRVC group died. The cause of clinical deterioration in the nonsurviving infants was clinical sepsis, with positive culture in one patient. Grade 3–4 IVH was detected in four of the infants who died (two in each group). 

## 4. Discussion

Mechanical ventilation is used when preterm infants have insufficient respiratory effort or in the presence of apnea or CO_2_ retention. CO_2_ excretion is determined by V_T_ and the respiratory rate. Volume-targeted ventilation (VTV) strategies aim to provide a consistent V_T_. VTV has been used for many years in pediatric and adult patients, and tidal volume-targeted synchronized ventilation modes are currently the most recommended strategy for ventilating preterm infants.

The bilevel VG mode and PRVC mode of the GE^®^ Carescape R860 model ventilator are two examples of adaptive target schemes for achieving target V_T_. This study aimed to compare and evaluate the safety and feasibility of these two modes for use in NICUs equipped with this ventilator model.

The literature includes very few studies of these modes and much less information about their use in neonates. 

In a study investigating the effects of PRVC and VG ventilation modes on oxygenation parameters, airway pressures, and immunomodulation during one-lung ventilation (OLV) in 70 adult patients undergoing thoracic surgery, oxygenation parameters (PaO_2_/FiO_2_ and PaO_2_) were significantly better in the PRVC group (*p* = 0.004). The dead space volume/tidal volume (V_D_/V_T_) and inspiratory airway pressures were significantly lower in the PRVC group. In addition, all inflammatory parameters (alveolar and plasma interleukins and alveolar albumin levels and cell counts) were significantly lower in the PRVC group. Therefore, it was concluded that the PRVC mode had positive effects during OLV in thoracic surgery [23].

Another study compared PCV and PRCV with VCV modes as lung-protective ventilation modes in 14 patients with acute lung injury or ARDS and examined whether PCV and PRVC reduced the work of breathing (WOB) more than VCV. A target V_T_ of 6.4 ± 0.5 mL/kg was specified during VCV and PRVC. During PCV, the inspiration pressure was adjusted as needed to achieve the same V_T_. The authors reported that PCV and PRVC provided no advantage over high-flow VCV in reducing WOB during lung-protective ventilation [24].

A study including 39 older intubated patients with chronic obstructive pulmonary disease compared PRVC and SIMV-VC modes and evaluated the efficacy of PRVC mode and its prevention of pulmonary barotrauma. Assessment of respiratory mechanics, arterial blood gas analysis, and vital signs at 2–4 h and at 48 h showed that PRVC resulted in rapid improvement in arterial blood gas parameters while maintaining low peak inspiratory pressure [25].

Kıhtır et al. evaluated 61 children (median age 12) with acute respiratory failure ventilated with PRVC or pressure-control (PC) modes. PC ventilation mode was used on 40 (65.6%) patients and PRVC mode was used on 21 (34.4%) patients. Forty-four patients (72.1%) had hypercapnic respiratory failure and twenty-eight patients (45.9%) had hypoxemic respiratory failure. Although the PC ventilation mode was preferred more frequently in hypoxemic respiratory failure, no difference was found in terms of MV duration, length of stay in the pediatric intensive care unit, and mortality between the two respiratory modes. It was stated that PRVC mode can be used as a safe option for units that do not have sufficient experience in using PC ventilation mode [26].

Another study compared PRVC and SIMV modes in 212 preterm infants under 1250 g who received mechanical ventilation in the first 6 h of life. There was no significant difference between the SIMV and PRVC groups in terms of infants alive and extubated on day 14 (41% vs. 37%), the duration of mechanical ventilation in survivors (median 24 vs. 33 days), or the proportion of infants not needing supplemental oxygen at postmenstrual 36 weeks (57% vs. 63%). Mode change due to unsuccessful ventilation was more frequent in the SIMV group (33%) than in the PRVC group (20%), but the inclusion of this unfavorable outcome did not change the overall result. The authors concluded that PRVC ventilation had no effect on the extubation time compared to SIMV [27].

In a study comparing PRVC and IMV modes in 60 neonates under 2500 g who required mechanical ventilation, the duration of ventilation and the incidence of BPD were found to be similar. The incidence of IVH grade 3 or higher was lower in the PRVC group (*p* < 0.005). In the subgroup analysis of infants under 1000 g, the PRVC group had shorter ventilation duration and a lower incidence of hypotension (*p* < 0.005). It was reported that PRVC ventilation can be used safely in newborns and may contribute to a lower complication rate [28].

Volume-targeted, volume-guaranteed ventilation is currently the most valid ventilation mode for preterm infants [4]. Some devices using VG are specially designed for neonates. VG provides better results in preterm infants in terms of BPD, PCO_2_ values, and air leaks. In our practice, we prefer VG and volume-targeted modes. We planned this study both because GE-brand ventilators have not been evaluated in neonatal and preterm infants in the medical literature and because these devices are available and being used in some hospitals.

In recent trials, valuable information regarding HFO ventilation in preterm infants has been provided. Although there is information that HFO can reduce BPD when used as the first mode in premature babies, more studies need to be designed on this subject [29,30].

To our knowledge, this is the first study comparing these two modes driven by the GE^®^ Carescape R860 model ventilator in very preterm infants. Moreover, the infants were diagnosed and treated by experienced staff with strictly adapted guidelines. 

The small number of patients is the main limitation of this study. Our results do not indicate that one of these modes is superior or more useful than other modes. However, devices and modes not previously used in neonatal studies were used as treatment options in very small preterm infants and were shown to be safe and applicable in this group. Another limitation may be our selection criteria because the infants included in the study are preterm infants with surfactant-treated RDS. The limited gestational age and the underlying cause may have altered the results. Larger studies including infants with different gestational ages and conditions that lead to intubation and invasive mechanical ventilation are required. 

## 5. Conclusions

In conclusion, both the PRVC and bilevel VG modes of GE ventilators can be used safely in preterm infants, and although the difference may not be very pronounced, bilevel VG mode was associated with more favorable early clinical findings. Studies including more patients and comparing them with other modes will clarify and provide further evidence on this subject.

## Figures and Tables

**Table 1:** Clinical characteristics and baseline values of the patients.

	Bilevel VG Group N = 14	PRVC Group N = 14	*p*
Birth weight, g, mean (SD)	890 (160)	861 (168)	0.642
Gestational age, weeks, mean (SD)	26.5 (1.3)	26.4 (1.6)	0.874
Sex, male, n (%)	8 (57)	7 (50)	0.704
Antenatal steroid, n (%)	12 (85)	11 (78)	0.621
Magnesium, n (%)	14 (100)	14 (100)	NS
Cesarean delivery, n (%)	10 (71)	11 (78)	0.662
Respiratory rate, /minute, median (range)	66 (46–78)	62 (55–78)	0.395
Heart rate, /minute, median (range)	144 (115–178)	140 (112–180)	0.973
Oxygen saturation, %, median (range)	89 (87–94)	90 (87–95)	0.398
Initial pH, median (range)	7.24 (7.12–7.40)	7.29 (7.1–7.45)	0.119
pCO_2_, median (range)	61 (38–70)	49 (37–67)	0.057
HCO_3_, median (range)	16 (13–19)	17 (14–21)	0.401
FiO_2_, %, median (range)	40 (25–60)	37 (25–60)	0.604
PIP, cmH_2_O, median (range)	15 (13–16)	16 (14–17)	0.041
MAP, cmH_2_O, median (range)	8 (7–9)	8 (7–9)	0.803
Compliance, mL/cmH_2_O, C_20_ (range)	1.65 (1.4–1.9)	1.7 (1.3–2)	0.517

VG: volume guarantee, PRVC: pressure-regulated volume control, SD: standard deviation, pCO_2_: partial pressure of carbon dioxide, FiO_2_: fraction of inspired oxygen, PIP: peak inspiratory pressure, MAP: mean airway pressure.

**Table 2:** Clinical outcomes.

	Bilevel VG Group N = 14	PRVC Group N = 14	*p*
Extubation time, days, median (range)	7 (5–16)	8 (5–20)	NS
PDA, n (%)	9 (64)	10 (71)	0.5
BPD (any stage), n (%)	10 (71)	10 (71)	0.661
ROP (any stage), n (%)	5 (35)	6 (42)	0.5
Sepsis, (clinical and proven) n (%)	5 (35)	7 (50)	0.352
IVH (stage 3–4), n (%)	2 (14)	2 (14)	NS
Mortality, n (%)	2 (14)	3 (21)	0.5

PDA: patent ductus arteriosus, BPD: bronchopulmonary dysplasia, ROP: retinopathy of prematurity, IVH: intraventricular hemorrhage, NS: non significant.

## Data Availability

The data that support the findings of this study are available from the corresponding author.
